# Chemokine-cleaving *Streptococcus pyogenes* protease SpyCEP is necessary and sufficient for bacterial dissemination within soft tissues and the respiratory tract

**DOI:** 10.1111/j.1365-2958.2010.07065.x

**Published:** 2010-02-10

**Authors:** Prathiba Kurupati, Claire E Turner, Ioanna Tziona, Richard A Lawrenson, Faraz M Alam, Mahrokh Nohadani, Gordon W Stamp, Annelies S Zinkernagel, Victor Nizet, Robert J Edwards, Shiranee Sriskandan

**Affiliations:** 1 Department of Infectious Diseases and Immunity, Imperial College London, Hammersmith HospitalDu Cane Road, London W12 0NN, UK; 2 Department of Experimental Medicine and Toxicology, Imperial College London, Hammersmith HospitalDu Cane Road, London W12 0NN, UK; 3 Histopathology Department, Imperial College London, Hammersmith HospitalDu Cane Road, London W12 0NN, UK; 4 Department of Pediatrics, University of California at San DiegoLa Jolla, CA 92093, USA

## Abstract

SpyCEP is a *Streptococcus pyogenes* protease that cleaves CXCL8/IL-8 and its activity is associated with human invasive disease severity. We investigated the role of SpyCEP in *S. pyogenes* necrotizing fasciitis and respiratory tract infection in mice using isogenic strains differing only in SpyCEP expression. SpyCEP cleaved human CXCL1, 2, 6 and 8 plus murine CXCL1 and 2 at a structurally conserved site. Mice were infected in thigh muscle with a strain of *S. pyogenes* that expresses a high level of SpyCEP, or with an isogenic non-SpyCEP expressing strain. SpyCEP expression by *S. pyogenes* hindered bacterial clearance from muscle, and enhanced bacterial spread, associated with cleavage of murine chemoattractant CXCL1. Mice were then infected with *Lactococcus lactis* strains that differed only in SpyCEP expression. In contrast to the parent *L. lactis* strain (lacks SpyCEP), which was avirulent when administered intramuscularly, infection with a strain that expressed SpyCEP heterologously led to dramatic systemic illness within 24 h, failure to clear bacteria from muscle and marked dissemination to other organs. In the upper airways, SpyCEP expression was required for survival of *L. lactis* but not *S. pyogenes*. However, dissemination of *S. pyogenes* to the lung was SpyCEP-dependent and was associated with evidence of chemokine cleavage. Taken together, the studies provide clear evidence that SpyCEP is necessary and sufficient for systemic bacterial dissemination from a soft tissue focus in this model and also underlies dissemination in the respiratory tract.

## Introduction

*Streptococcus pyogenes* causes a range of invasive infections including necrotizing fasciitis and myonecrosis. Despite an attendant mortality as high as 50% (Stevens, 1995), the pathogenesis of *S. pyogenes* necrotizing fasciitis is not completely understood. Many of the systemic features of profound septic shock that commonly accompany necrotizing fasciitis may stem from bacterial release of exotoxins, including superantigens (Cunningham, 2000). The ability of *S. pyogenes* to spread rapidly at the site of infection and to disseminate systemically indicates the pathogen possesses robust mechanisms to resist the human innate immune response.

*Streptococcus pyogenes* co-ordinates an array of virulence factors to combat host opsonophagocytosis (Nizet, 2007). The streptococcal interleukin-8 (CXCL8/IL-8) inactivating cell envelope protease, SpyCEP/*cepA* (Edwards *et al.*, 2005) [genomic annotation *prtS*, also known as *scpC* (Ferretti *et al.*, 2001; Hidalgo-Grass *et al.*, 2006)], is markedly upregulated in *S. pyogenes* strains during the transition to invasive infection (Sumby *et al.*, 2006; 2008; Turner *et al.*, 2009a). Coupled with clinical observations that correlate SpyCEP production to disease severity (Turner *et al.*, 2009a), the published data suggest a central role for SpyCEP in invasive infection.

SpyCEP cleaves ELR motif-positive CXC chemokines such as CXCL8/IL-8, CXCL1/Gro-α and CXCL6/GCP-2, as well as murine CXCL1/KC and CXCL2/MIP2 (Edwards *et al.*, 2005; Hidalgo-Grass *et al.*, 2006; Sumby *et al.*, 2008; Fritzer *et al.*, 2009). These chemokines are known to signal via CXCR1 and CXCR2 receptors on phagocytes to activate and recruit such cells to the site of infection. SpyCEP cleaves CXCL8/IL-8 within its C-terminal α-helix, resulting in diminished function (Edwards *et al.*, 2005); other than murine CXCL2/MIP-2, the precise cleavage sites within other chemokines have, to date, not been defined.

A number of investigators have sought to ascribe a role for SpyCEP in soft tissue infection by assessment of lesion size, yet the findings have been inconsistent, and careful analysis of key parameters, such as bacterial load, clearance and systemic spread, has yet to be performed (Hidalgo-Grass *et al.*, 2006; Sjolinder *et al.*, 2008; Sumby *et al.*, 2008; Zinkernagel *et al.*, 2008). In this study we set out to explore the specific role of SpyCEP in necrotizing soft tissue infection, with a critical focus on bacterial clearance and bacterial dissemination. We couple loss-of-function analysis, via an isogenic SpyCEP-deficient mutant created in a strain of *S. pyogenes* derived from a patient with necrotizing fasciitis, with gain-of-function analysis, through heterologous expression of SpyCEP in *Lactococcus lactis*. The data unequivocally demonstrate that SpyCEP expression impedes bacterial clearance and augments dissemination of bacteria, both to the regional lymph node, and into the systemic circulation. Infection with SpyCEP-expressing *L. lactis* reproduced many of the salient features of severe necrotizing *S. pyogenes* infection. Failure of neutrophil-mediated bacterial clearance orchestrated by the single virulence factor SpyCEP may be a pivotal determinant of tissue necrosis and lethality observed in severe *S. pyogenes* infection.

## Results

### SpyCEP cleaves CXCL1, CXCL2, CXCL6 and CXCL8 at a structurally conserved site

**Fig. 1:**
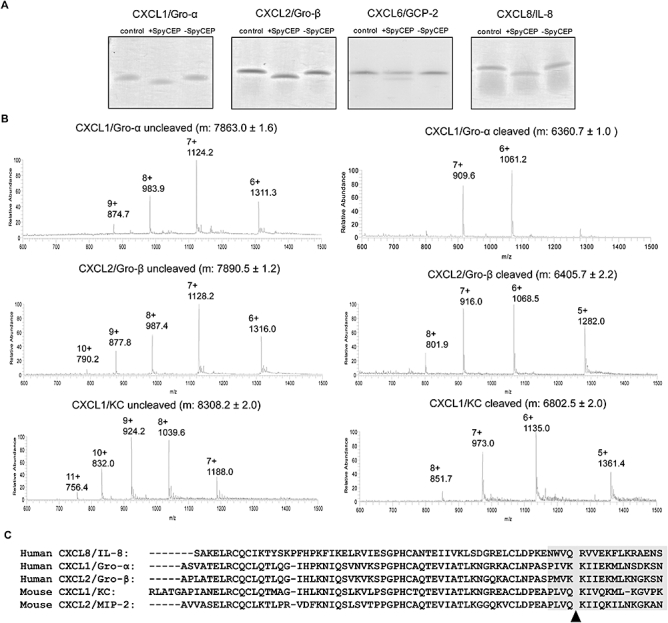
SpyCEP cleaves human CXCL1, 2, 6 and 8 and murine CXCL1/2 after the first four amino acids of the C-terminal α-helix. A. Colloidal Coomassie blue stained SDS-PAGE gels showing human chemokines cleaved by SpyCEP. Chemokines were co-incubated either alone (left lane); with supernatant containing SpyCEP, H292 (+SpyCEP, middle lane); or supernatant without SpyCEP, H575 (-SpyCEP, right lane). B. Mass spectroscopy analysis of uncleaved and cleaved chemokines using electrospray ionization generated a series of multiply charged ions (indicated as m/z; mass-to-charge ratio) from which the average molecular mass (m) of each was deduced. C. Site of SpyCEP-mediated cleavage of various chemokines determined in this study and previously for CXCL8/IL-8 and CXCL2/MIP-2 (Edwards *et al.*, 2005): arrowhead. The position of the α-helix in this region is indicated by a grey shaded box.

The susceptibility of a wide panel of human CXC chemokines (CXCL1-CXCL12) to cleavage by SpyCEP was systematically investigated by SDS PAGE analysis. Four ELR motif-containing human chemokines (CXCL1/Gro-α, CXCL2/Gro-β, CXCL6/GCP-2, and CXCL8/IL-8) were cleaved, all of which are known to act as ligands for CXCR1 and CXCR2 (Fig. 1A). Other human CXC chemokines were not cleaved (not shown). The cleavage sites of CXCL8/IL-8 and murine CXCL2/MIP-2 were previously reported (Edwards *et al.*, 2005). Mass spectrometry analysis of cleaved and uncleaved human CXCL1/Gro-α and CXCL2/Gro-β identified the SpyCEP cleavage site to be between lysine residues K_60_ and K_61_ in both chemokines, while analysis demonstrated the murine CXCL1/KC molecule to be cleaved between Q_65_ and K_66_. (Fig. 1B and C). In each case, cleavage occurred just distal from the known or predicted start of the C-terminal α-helix regardless of differences in amino acid residues flanking the cleavage site, suggesting that SpyCEP cleaves the ELR motif CXC chemokines at a site that is structurally conserved. The cleavage site of CXCL6/GCP-2, which demonstrated only partial cleavage, could not be defined with certainty.

### SpyCEP resists bacterial clearance and contributes to *S. pyogenes* dissemination from soft tissue

**Fig. 2:**
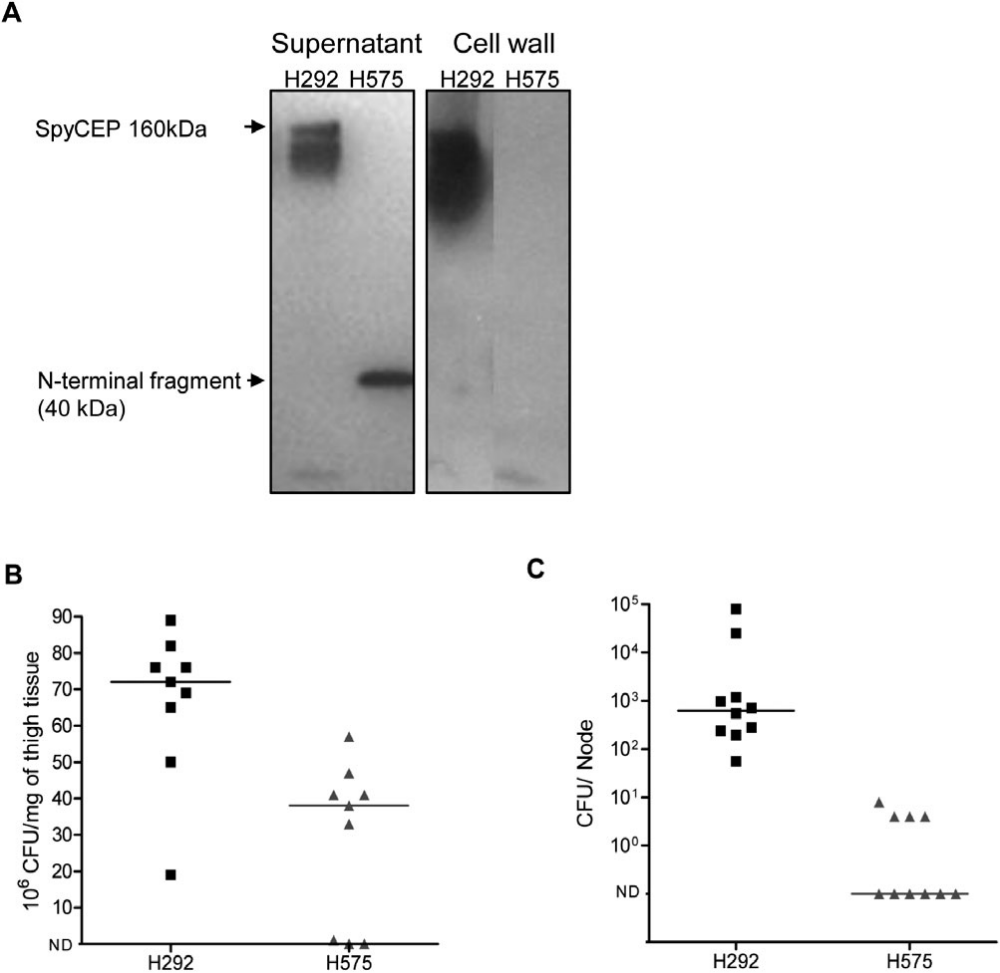
SpyCEP influences bacterial clearance in soft tissue and systemic spread of *S. pyogenes*. A. SpyCEP production by *S. pyogenes* parent strain H292 and mutant *cepA* strain H575. Western blots were performed on culture supernatants (left panel) and cell wall preparations (right panel). Wild-type SpyCEP was detected as an approximate 160 kDa band in both the supernatant and the cell wall preparation from strain H292. Strain H575 produced an inactive truncated N-terminal SpyCEP fragment of approximately 40 kDa in the culture supernatant, but not the cell wall due to lack of a cell wall anchor motif. B. Expression of SpyCEP by strain H292 enhanced *S. pyogenes* survival at the site of infection following intramuscular infection of CD1 female mice compared with strain H575 (*P* = 0.0019; inoculum 2 × 10^8^ cfu). C. Expression of SpyCEP also enhanced spread of *S. pyogenes* to the regional lymph node (*P* = 0.0001). *n* = 10 mice per group in each experiment, representative of two experiments. ND, not detected; cfu, colony-forming units; horizontal line, median.

We sought to determine the role of SpyCEP in invasive infection using a clinical necrotizing fasciitis strain that expresses SpyCEP at high level, as high level expression of SpyCEP characterizes many invasive infections (Turner *et al.*, 2009a). In comparison to the parent *S. pyogenes* strain (H292), the mutant *cepA* strain (H575) produced a C-terminally truncated SpyCEP protein that lacked one of the three residues necessary for serine protease activity (Fig. 2A). Functional disruption of the *cepA* locus was confirmed by Southern blotting (Fig. S1A) and CXCL8/IL-8 cleavage studies, which demonstrated unequivocally that SpyCEP was responsible for CXCL8/IL-8 cleavage (Fig. S1B). Strains H575 and H292 were phenotypically similar and demonstrated no differences in growth in broth or whole blood. Proteomic analysis of secreted proteins demonstrated no difference between H575 and H292 except for the production of a truncated SpyCEP protein by H575, and production of the cysteine protease SpeB was unchanged between the two strains. To determine whether the insertion mutation led to polar effects on surrounding genes, real-time PCR analysis was conducted on the two genes immediately upstream of *cepA* (*exoA* and *lctO*) and four genes immediately downstream of *cepA* (hypothetical permease, *metS*, *nrdF*, *nrdE*). There were no differences in expression of these genes in H575 compared with H292 during either exponential or early stationary growth phase, suggesting that the mutation of *cepA* did not result in disruption of the surrounding genes (data not shown).

Murine intramuscular infection with *S. pyogenes* parent and *cepA* mutant strains demonstrated that host clearance of *S. pyogenes* from muscle at 24 h after infection onset was impeded by SpyCEP expression (Fig. 2B), providing the first evidence that SpyCEP interferes with bacterial clearance at the site of deep soft tissue infection. Bacterial dissemination to the regional draining lymph node was augmented 100- to 1000-fold by SpyCEP; indeed, in the absence of SpyCEP, mice demonstrated little or no dissemination of *S. pyogenes* (Fig. 2C). Dissemination to other organs or blood was not observed at the bacterial inocula used, so the inoculum was increased to provide a 10-fold higher dose (10^9^ colony-forming units, cfu). Mice infected with wild-type *S. pyogenes* had a significantly greater bacterial load in blood (median, 1 × 10^3^ cfu ml^−1^; range, 3 × 10^2^ to 1 × 10^5^ cfu ml^−1^) than mice infected with the *cepA* mutant strain H575 (median, undetectable; range, undetectable to 3 × 10^2^ cfu ml^−1^; *P* < 0.01).

### Cleavage of murine CXCL1/KC locally and systemically by SpyCEP during *S. pyogenes* infection

**Fig. 3:**
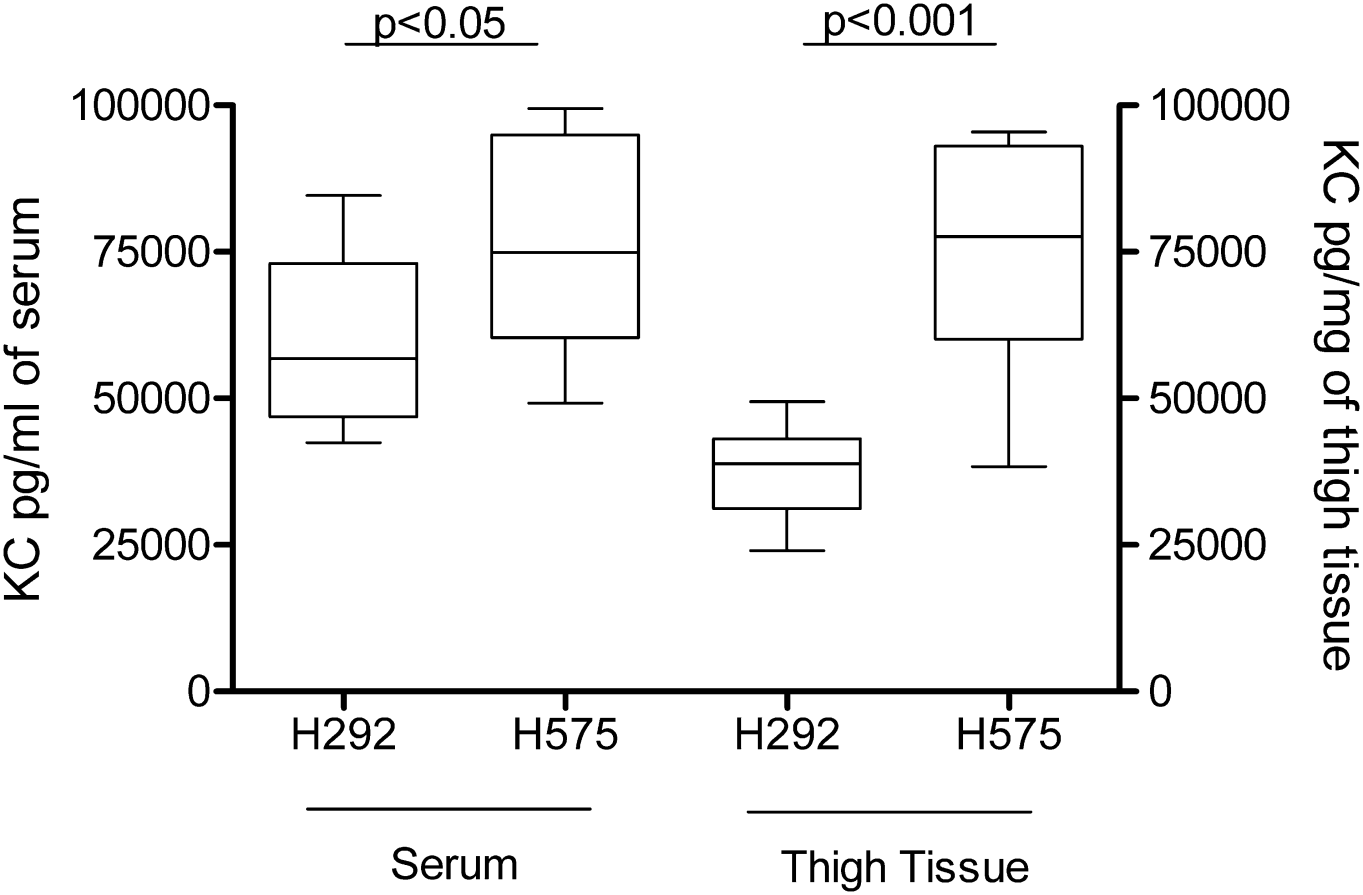
Levels of KC (murine CXCL1) in mouse serum (left axis) and mouse whole thigh homogenate (right axis) 24 h after infection with *S. pyogenes*. KC was measured by ELISA. Infection with parent strain H292 reduced chemokine levels compared with those mice infected with mutant strain H575 in both serum and thigh muscle. *n* = 10 mice per group.

Chemokine levels normally reflect bacterial load during *S. pyogenes* infection. Despite an increased bacterial burden, infection with wild-type *S. pyogenes* actually reduced CXCL1/KC levels in muscle tissue (median, 37 ng ml^−1^; range, 23–49 ng ml^−1^) compared with mice infected with the *cepA* mutant (median, 78 ng ml^−1^; range, 37–93 ng ml^−1^; *P* < 0.001), consistent with chemokine inactivation by SpyCEP (Fig. 3), since the enzyme-linked immunosorbant assay (ELISA) recognizes only full-length rather than cleaved CXCL1/KC. Notably, inactivation of CXCL1/KC by SpyCEP extended beyond the site of infection, affecting serum CXCL1/KC levels in mice infected with wild-type *S. pyogenes* (median, 57 ng ml^−1^; range, 43–84 ng ml^−1^) compared with those infected with the *cepA* mutant (median, 75 ng ml^−1^; range, 48–99 ng ml^−1^; *P* < 0.05) (Fig. 3).

### Expression of SpyCEP in *L. lactis* confers ability to resist bacterial clearance from soft tissue and disseminate systemically

**Fig. 4:**
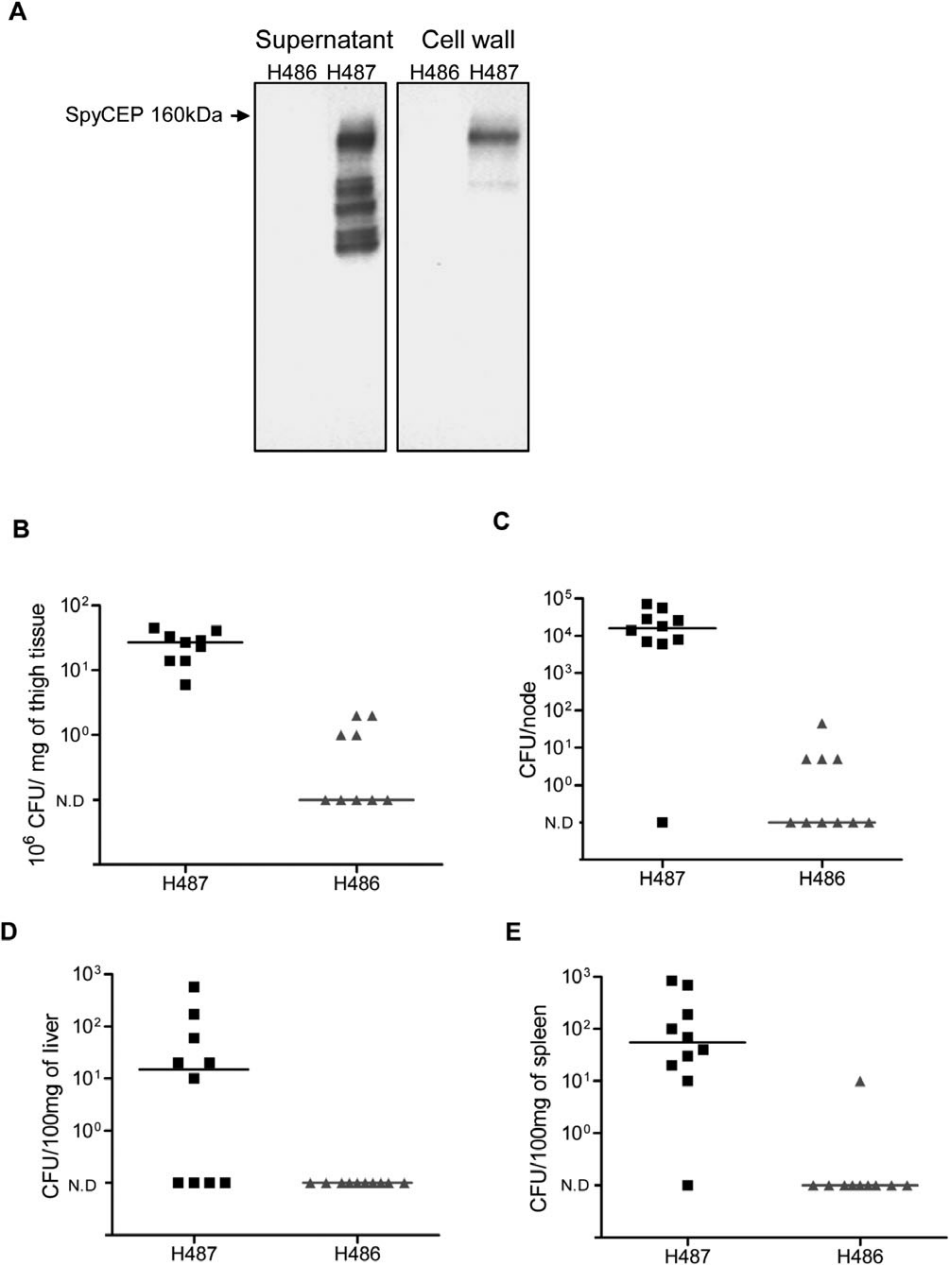
Heterologous expression of SpyCEP in *L. lactis* is sufficient to resist bacterial clearance and promote dissemination. A. Heterologous expression of SpyCEP by *L. lactis* strain H487 and parental control *L. lactis* strain H486. Western blots for SpyCEP were performed on culture supernatants (left panel) and cell wall preparations (right panel). H487 produced SpyCEP in both supernatant and cell wall. Lower molecular weight bands represent autocatalytic cleavage of SpyCEP. B. Bacterial quantification in muscle tissue demonstrating heavy growth in mice infected with SpyCEP-expressing *L. lactis* strain H487 compared with control strain H486 (*P* = 0.0001). C. Bacterial dissemination of *L. lactis* to regional lymph node. Quantification of bacteria in each inguinal lymph node shows greater bacterial spread to node in mice infected with H487 compared with H486 (*P* = 0.0015). D. Bacterial quantification in liver. SpyCEP was also essential for systemic spread of *L. lactis* to liver with greater bacterial burden in liver of mice infected with H487 compared with H486 (*P* = 0.012). E. Bacterial quantification in spleen with greater bacterial burden in spleen of mice infected with H487 compared with H486 (*P* = 0.0003). *n* = 10 per group representative of two experiments. ND, not detected; cfu, colony-forming units; horizontal line, median.

*Lactococcus lactis* is a non-pathogenic bacterium that does not produce SpyCEP, nor cleave CXCL8/IL-8 and related chemokines. However, *L. lactis* strain H487 was engineered to heterologously express biologically active SpyCEP (Zinkernagel *et al.*, 2008). Western blotting confirmed that SpyCEP was produced both at the cell wall and also as a soluble exoprotein, released into culture supernatant, emulating SpyCEP production by *S. pyogenes* (Fig. 4A). The concentration of SpyCEP released by H487 into the culture supernatant was measured by ELISA and was found to be 12 ng ml^−1^ compared with 69 ng ml^−1^ made by H292. Being avirulent, *L. lactis* is normally rapidly cleared by the murine immune system (Que *et al.*, 2005; Maisey *et al.*, 2008); indeed, in pilot experiments using a high inoculum of 10^11^ cfu, *L. lactis* could not be detected in muscle 24 h after intramuscular administration. In contrast, following intramuscular infection with *L. lactis*, heterologous expression of SpyCEP by *L. lactis* conferred the ability to resist clearance at the site of infection (median, 5 × 10^7^ cfu mg^−1^; range, 8 × 10^6^ to 7 × 10^7^ cfu mg^−1^) compared with the parent lactococcal strain (median, undetectable; range, undetectable to 4 × 10^6^ cfu ml^−1^; *P* = 0.0001) (Fig. 4B). Heterologous expression of SpyCEP also permitted bacterial dissemination to regional lymph node, liver and spleen (Fig. 4C–E). In separate experiments conducted to detect bacteremia, all mice infected with SpyCEP-expressing *L. lactis* intramuscularly were bacteremic at 24 h (median 280 cfu ml^−1^, range 192 to 1 × 10^7^ cfu ml^−1^), whereas bacteremia was not detected in any mouse infected with the parent *L. lactis* strain.

The chemokine CXCL1/KC could not be detected at all in mice infected with the parent *L. lactis* strain, consistent with their complete recovery and clearance of bacteria, thus it was not possible to compare chemokine levels between groups.

### Heterologous expression of SpyCEP in *L. lactis* is sufficient to cause soft tissue necrosis, systemic disease and death

Although the primary aim of the study was to obtain quantitative bacteriological data, 10/10 mice infected with SpyCEP-expressing *L. lactis* reached the protocol-defined survival end-point for infection by 24 h (unresponsive to stimuli, loss of spontaneous movement), whereas all mice infected with the parent *L. lactis* strain showed no ill effects. Furthermore, mice infected with SpyCEP-expressing *L. lactis* lost more weight (median 93% of original weight, range 88.8–98.9%) during the 24 h experiment than those infected with the parent *L. lactis* strain (median 100.6% of original weight, range 97.7–103%, *P* < 0.0001). Taken together, the above data demonstrate that SpyCEP conferred a lethal phenotype to *L. lactis* that was manifest within 24 h of infection.

**Fig. 5:**
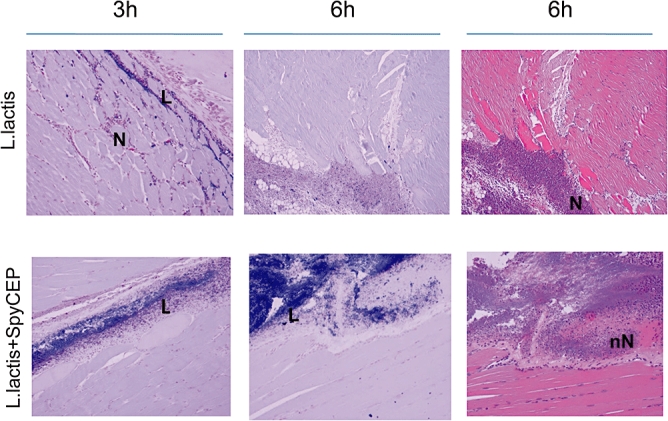
Tissue sections from thigh muscle of *L. lactis*-infected mice obtained at 3 and 6 h after onset of infection suggest that SpyCEP is sufficient to reproduce some features of invasive necrotizing infection. Gram stained muscle sections from *L. lactis*-infected mice at 3 and 6 h after infection (left and central panels). Upper panels, mice infected with control *L. lactis* H486 (empty plasmid); Lower panels, mice infected with *L. lactis* strain expressing SpyCEP, H487. Right hand panels, haematoxylin and eosin-stained tissues at 6 h. L, lactococci; N, neutrophils; nN, necrotic neutrophil. Representative of three mice at each time point, magnification ×200.

Blood samples taken from mice 7 h after *L. lactis* infection demonstrated that mice infected with SpyCEP expressing strains were already bacteremic (median 448 cfu ml^−1^, range 28–764 cfu ml^−1^), whereas bacteremia was not seen in mice infected with the parent *L. lactis* strain. To investigate early pathogenic events, histopathology specimens from the thigh were obtained 3 and 6 h following infection with *L. lactis*. Studies demonstrated that visible differences in bacterial load between experimental groups of mice evolved between 3 and 6 h after administration of bacterial inocula (Fig. 5). At 6 h, confluent areas of Gram-positive bacterial growth were observed in mice infected with the SpyCEP-expressing *L. lactis* in contrast to mice infected with the wild-type *L. lactis*, where bacteria could not be detected. Despite expression of SpyCEP, inflammatory cells were visible at the site of infection in mice infected with the SpyCEP-expressing *L. lactis* strain. A visible necrotic inflammatory infiltrate was observed in tissues of mice exposed to SpyCEP-expressing *L. lactis* around areas of bacterial confluent growth, despite the fact that *L. lactis* itself does not express virulence factors known to degrade mammalian tissues or elicit neutrophil necrosis. The unopposed spread of bacteria along tissue planes was very similar to that seen in aggressive *S. pyogenes* necrotizing fasciitis (Sriskandan *et al.*, 1999).

### SpyCEP enhances *S. pyogenes* dissemination to lower respiratory tract from nasopharynx

**Fig. 6:**
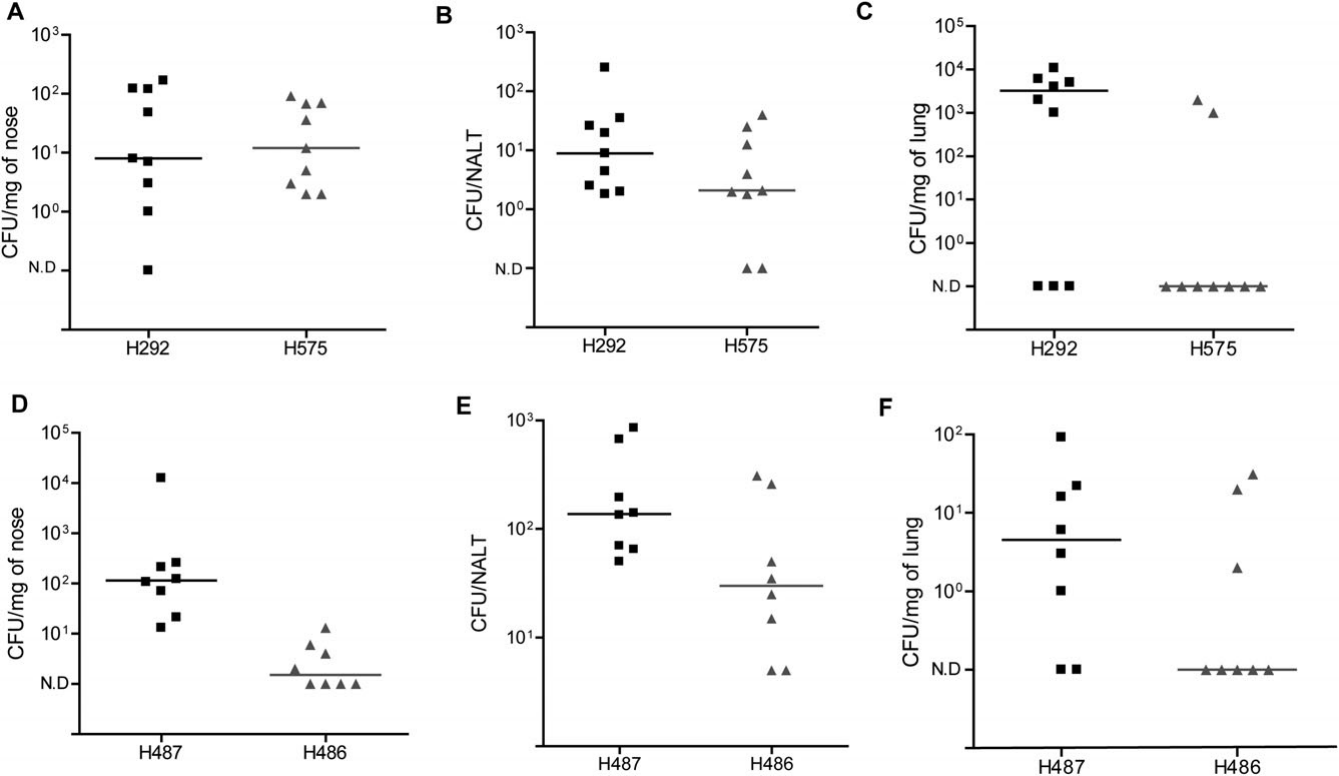
SpyCEP enhances *S. pyogenes* dissemination to lung from the upper respiratory tract. *S. pyogenes* studies are shown in (A), (B) and (C) (10 mice per group). Following intranasal infection, *S. pyogenes* survival in the nasal tissues (A) and NALT (B) was unaffected by disruption of SpyCEP. However, *S. pyogenes* dissemination into the lower respiratory tract (C) was enhanced by SpyCEP expression (*P* = 0.02). Lactococcal studies are shown in (D), (E) and (F) (8 mice per group). SpyCEP expression by *L. lactis* was sufficient to allow survival in the nasal tissues (*P* = 0.001) (D) and also enhanced survival in NALT (*P* = 0.046) (E). SpyCEP did not significantly affect survival of *L. lactis* in the lower respiratory tract (F). ND, not detected; cfu, colony-forming units; horizontal line, median. Data from one experiment for each strain.

Following intranasal infection with *S. pyogenes* or with *L. lactis*, *S. pyogenes* could be recovered from the nose and nasal associated lymphoid tissues (NALT) 24 h after administration (Fig. 6A and B), in contrast to *L. lactis,* which was cleared by 24 h (Fig. 6D and E). Although SpyCEP expression did not measurably contribute to survival of *S. pyogenes* in the nasopharynx, heterologous expression of SpyCEP was sufficient to allow survival in the nasopharynx by *L. lactis* (Fig. 6A, B, D and E). Intriguingly, however, SpyCEP expression did enhance *S. pyogenes* dissemination to the lung from the nasopharynx although it was not, alone, sufficient to allow *L. lactis* to survive in the lung (Fig. 6C and F). Mice infected with the parent *S. pyogenes* strain demonstrated higher bacterial counts in the lung (median, 2 × 10^3^ cfu mg^−1^; range, undetectable to 1 × 10^4^ cfu mg^−1^), than mice infected with the non-SpyCEP producing mutant (median, undetectable; range, undetectable to 2 × 10^3^ cfu mg^−1^).

### SpyCEP leads to local and systemic chemokine cleavage during *S. pyogenes* lower respiratory tract infection

**Fig. 7:**
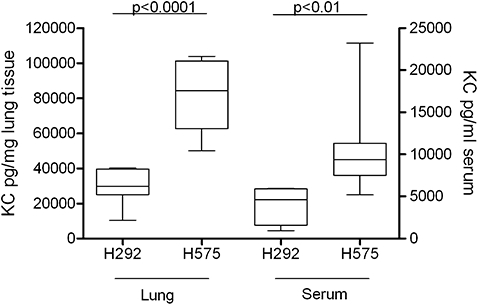
Levels of KC (murine CXCL1) in mouse lung homogenate (left side axis) and mouse serum (right side axis) 24 h after infection with *S. pyogenes*. Infection with parent strain H292 reduced chemokine levels compared with those mice infected with mutant strain H575 in both lung and serum. *n* = 10 mice per group.

In mice infected with *S. pyogenes*, there was an inverse relationship between bacterial load and CXCL1/KC levels during respiratory tract infection. Despite higher bacterial loads, mice infected with the parent *S. pyogenes* strain demonstrated lower levels of CXCL1/KC in both lung tissue and serum (lung median, 32 ng ml^−1^; range, 10–40 ng ml^−1^; serum median, 4.7 ng ml^−1^; range, 1–6 ng ml^−1^) than mice infected with the non-SpyCEP producing mutant (lung median, 86 ng ml^−1^; range, 50–120 ng ml^−1^, *P* < 0.0001; serum median, 9 ng ml^−1^; range, 5–23 ng ml^−1^, *P* < 0.01) (Fig. 7). This is consistent with chemokine cleavage occurring both in the lung and systemically as a specific effect of SpyCEP.

## Discussion

SpyCEP is a recently described serine protease of *S. pyogenes* that cleaves the major human chemokine CXCL8/IL-8 through the origin of the C-terminal α-helix (Edwards *et al.*, 2005). Clinical and transcriptomic studies suggest that SpyCEP may play a central role in invasive *S. pyogenes* infection (Sumby *et al.*, 2006; Sumby *et al.*, 2008; Turner *et al.*, 2009a). In this study, we sought to define the role played by SpyCEP in both soft tissue and lower respiratory tract invasive infection, using both site-directed mutagenesis of *S. pyogenes* and heterologous expression in *L. lactis*.

We demonstrate the cleavage sites of each SpyCEP-cleaved chemokine to be restricted to a single site within the first four bases of the predicted C-terminal α-helix. Taken together with the previously reported cleavage sites of CXCL8/IL-8 (Q_59_–R_60_) and murine CXCL2/MIP-2 (Q_60_–K_61_) (Edwards *et al.*, 2005), the sites for cleavage by SpyCEP appear to be highly specific and dictated by location within the tertiary structure of the protein rather than by a simple sequence motif.

Phenotypic changes in *S. pyogenes* can arise during invasive soft tissue infection, and have been linked in some cases to genotypic changes in the two component virulence regulator, *covR/S* (Engleberg *et al.*, 2001; Sumby *et al.*, 2006; Walker *et al.*, 2007). Although *covR/S* regulates many genes necessary for resistance to opsonophagocytosis, the invasive phenotype is characterized, in particular, by increased transcription of the gene encoding SpyCEP, suggesting a central role for SpyCEP in invasive infection (Sumby *et al.*, 2006; 2008; Turner *et al.*, 2009a). In the current study we determined the role of SpyCEP in invasive infection using a clinical necrotizing fasciitis *S. pyogenes* strain, H292, that expresses SpyCEP at high level (Turner *et al.*, 2009a). In this strain, SpyCEP was necessary for systemic dissemination, exerting a marked effect on spread to lymph node from soft tissue. We were unable to complement the isogenic mutant strain H575 but studies suggested that the mutation had not resulted in a polar or non-specific effect on phenotype. Three other recent studies have demonstrated that SpyCEP contributes to increased lesion size in streptococcal dermal necrosis (Hidalgo-Grass *et al.*, 2006; Sjolinder *et al.*, 2008; Zinkernagel *et al.*, 2008), although a similar mutation in a different strain background did not produce this pattern of virulence attenuation (Sumby *et al.*, 2008). Differences between mouse strains, bacterial strains, baseline SpyCEP expression, or *covR/S* mutations arising during infection, may account for the varied findings.

In the upper respiratory tract, SpyCEP contributed to nasopharyngeal *S. pyogenes* survival but was not essential. *S. pyogenes* expresses a number of virulence factors that may allow infection or colonization of the nasopharynx, thereby compensating for SpyCEP. In contrast, SpyCEP expression played a greater role in the spread of *S. pyogenes* from nasopharynx to lung. This is relevant to the pathogenesis of *S. pyogenes* lower respiratory tract infection, a common clinical focus in addition to skin and soft tissue disease. Given that regulatory changes affecting SpyCEP expression are hypothesized to occur during the transition from pharyngeal to invasive infection (Sumby *et al.*, 2006), the findings may also be relevant to the systemic dissemination of *S. pyogenes* from the upper respiratory tract.

Heterologous expression of SpyCEP by the otherwise avirulent *L. lactis* led to a striking illness associated with a failure to clear bacteria from soft tissue and dissemination of bacteria to lymph node, distant organs and blood. ELISA showed that SpyCEP production by the transformed *L. lactis* was not greater than the wild-type *S. pyogenes* strain, thus it is unlikely that the results obtained arose due to excessive production of SpyCEP compared with *S. pyogenes* (Figs 2A and 3A).

To further understand the reasons for uncontrolled sepsis in *L. lactis*-infected mice, histopathological examination of infected muscle was undertaken, confirming that the effects of SpyCEP are manifest as early as 3–6 h after infection onset. The features of confluent Gram-positive bacteria spreading along tissue planes associated with necrotic inflammation were similar to those observed in necrotizing *S. pyogenes* infection. The findings raise the possibility that the features of necrotizing soft tissue infection arise directly from host cell exposure to SpyCEP or through exposure to an inadequately cleared necrotic inflammatory infiltrate. Our preliminary studies do not support a role for SpyCEP in enzymatic cleavage of mammalian proteins other than chemokines (P.K., C.T., S.S., unpublished). Histopathology studies demonstrated that neutrophil recruitment was not completely inhibited in mice infected with SpyCEP-expressing *L. lactis*. We considered the possibility that partial loss of the SpyCEP expression plasmid may occur during infection. Experiments demonstrated that erythromycin resistance was maintained in both isogenic *L. lactis* strains at all time points studied, thus plasmid loss was unlikely to affect chemokine cleavage. We conclude that some neutrophil recruitment will occur either independent of CXC chemokines or through residual chemokine activity. Despite the presence of inflammatory cells, expression of SpyCEP by *L. lactis* successfully prevented bacterial clearance. This is consistent with the hypothesis that SpyCEP confers additional harm to neutrophil function and may prevent neutrophil killing of bacteria. Inactivation of CXCR1 and 2 ligands will have pleiotropic effects on neutrophil function and may additionally influence neutrophil death pathways (Guichard *et al.*, 2005).

SpyCEP is a member of a larger family of cell envelope proteases homologous to lactococcal enzymes that cleave casein (Siezen, 1999). Among pathogenic streptococci, these genes have diversified to yield a family of *s*treptococcal *C*5a *p*eptidases (*scpA* and *B*), which do not cleave chemokines (Hill *et al.*, 1988; Cleary *et al.*, 1992), and a separate family of CXC chemokine-cleaving cell envelope proteinases (CEPs), which do not cleave C5a (Harris *et al.*, 2003; Zinkernagel *et al.*, 2008; Bryan and Shelver, 2009; Turner *et al.*, 2009b). The pathogenic impact of this wider family of chemokine cleaving enzymes in other hemolytic streptococci remains to be fully explored.

SpyCEP impacts on neutrophil recruitment, phagocytic function and longevity (Edwards *et al.*, 2005; Sumby *et al.*, 2008; Zinkernagel *et al.*, 2008); it may also have effects on neutrophil clearance and the relative contributions of these different functions to pathogenesis are the subject of further study. Active immunity against SpyCEP halts dissemination of *S. pyogenes* during soft tissue infection (Turner *et al.*, 2009b), suggesting that SpyCEP may be a suitable vaccine target for protection against invasive *S. pyogenes* infection. Together with the current data, it is evident that SpyCEP plays a central role in bacterial dissemination during invasive *S. pyogenes* infection, conferring a lethal phenotype that may contribute to the rapid clinical deterioration so often observed in patients with invasive streptococcal infection.

## Experimental procedures

### Bacterial strains and construction of a SpyCEP mutant

*Streptococcus pyogenes* invasive isolate H292 (*emm*81) was isolated from a patient with bacteremia and lethal necrotizing fasciitis and was the parent strain used to create a Δ*cepA* knock-out. *L. lactis* expressing SpyCEP (H487) and *L. lactis* containing empty plasmid pDESTerm (H486) were reported previously (Zinkernagel *et al.*, 2008). *S. pyogenes* and *L. lactis* were grown in Todd-Hewitt broth (Oxoid, Basingstoke, UK) or on Todd-Hewitt agar or Columbia horse blood agar. *Escherichia coli* strains were cultured in Luria–Bertani broth (Oxoid). Antibiotics were used at the following concentrations: erythromycin 1 µg ml^−1^ (for *S. pyogenes*) or 5 µg ml^−1^ (for *L. lactis*), and kanamycin 50 µg ml^−1^ for *E. coli*.

The SpyCEP gene, *cepA*, was disrupted by site-directed mutagenesis in *S. pyogenes* strain H292 to generate strain H575. Briefly, a 246–974 bp region of the *cepA* coding sequence was amplified using *cepA*-knock-out primers *cepA*F (CGGAATTCAACCACAACGAGTGAACCAA) and *cepA*R (CGGAATTCTGCATACCGTGTGACTCGTAT) from strain H292 DNA and cloned into the EcoRI site of the temperature-sensitive shuttle vector pGHost*aph1* (Unnikrishnan *et al.*, 2002). The plasmid, designated pGHost-*cepA,* was extracted from *E. coli* and then used to transform *S. pyogenes* strain H292 by electroporation. Transformants were selected using erythromycin at 30°C and then moved to 37°C in liquid culture to obtain plasmid integration. Transformants were analysed by Southern and Western blot to confirm disruption of *cepA*. Isogenic isolates were compared with regard to CXCL8/IL-8 cleaving activity, by sodium dodecyl sulphate polyacrylamide gel electrophoresis (SDS-PAGE). Isolates were also compared by SDS-PAGE analysis of secreted proteins, and by expression of the cysteine protease, SpeB by western analysis using polyclonal antiSPEB antibody (Toxin Technology, Sarasota, FL, USA).

### Southern and Western hybridization

Southern analysis was performed on XmnI-cut genomic DNA extracted from the parent strain H292 and transformant, H575, using a 728 bp digoxygenin (DIG)-labelled *cepA* probe (amplicon prepared using primers *cepA*F and *cepA*R). Southern blot of XmnI-cut genomic DNA demonstrated a change in the *cepA* locus from 4.9 kb in H292 to 6.5 kb in H575 confirming pGHost-*cepA* integration (Fig. S1B).

Western blot analysis of culture supernatant and cell wall fractions was performed as described previously (Turner *et al.*, 2009a). Briefly, cell wall fraction was obtained after 3 h incubation of cell pellets with 10 mM Tris-HCl, 30% raffinose, 100 U ml^−1^ mutanolysin, 1 mg ml^−1^ lysozyme and Calbiochem protease inhibitor III. Cell wall fractions were then collected in the resulting supernatant. Proteins were separated using pre-cast 10% Bis-Tris SDS-PAGE gels (Invitrogen, UK) and immunoblotted with a rabbit polyclonal anti-SpyCEP serum raised against a recombinant protein representing residues 35–587 of the pre-pro SpyCEP enzyme sequence (GenBank DQ413032) (Turner *et al.*, 2009b). Blots were developed using the ECL system (GE Healthcare, UK).

### Real-time PCR analysis

Primers were designed to amplify a 100–200 bp region of each of the genes surrounding the *cepA* locus; exoribonuclese III (*exoA*, exoA F: 5′-CATCACCAGCATTAGGCGTG-3′, exoA R: 5′-CTATGCTGGCACCATGTTCC-3′) and lactate oxidase (*lctO*, lctO F: 5′-GGAAGTATCTACACCACTAGTTCC-3′, lctO R: 5′-GCTTTGACACGATCCATAAT-3′) upstream and a hypothetical permease (Permease F: 5′-GTCAGAACAGCAACTGAAGG-3′, Permease R: 5′-AGCTGCTACCGTAACATTGG-3′), methionyl-tRNA synthetase (*metG/S*, metS F: 5′-GAAGACGGTCAGGTTATTGG-3′, metS R: 5′-AGTACGGCTAACAGCCAAATC-3′), ribonucleoside-diphosphate reductase β-chain (*nrdF*, NrdF F: 5′-TTTACCTATCAGCACGTGG-3′, NrdR R: 5′-TGCATACAGTTCTCTCAAGTAAGC-3′), and ribonucleoside-diphosphate reductase α-chain (*nrdE.2*, NrdE F: 5′-CTGCTAGCCAATCCAGCCATC-3′, NrdE R: 5′-CACAGGTTAGACATGACGATTC-3′) downstream. RNA was extracted from H292 and H575 at exponential phase (A_600_ 0.5) and early stationary phase (A_600_ 0.7–0.8) and converted into cDNA as described previously (Turner *et al.*, 2009a). Real-time PCR was performed using SYBR green as described previously (Turner *et al.*, 2009a) and normalized to the housekeeping gene *gyrA*. Relative gene expression and statistical analysis were performed using REST analysis (Pfaffl *et al.*, 2002).

### SpyCEP enzyme-linked immunosorbant assay

Anti-SpyCEP antibodies were purified from rabbit polyclonal anti-SpyCEP serum using CnBr-activated sepharose 4 fast flow (GE Healthcare). Purified antibody (2 mg ml^−1^) was labelled with biotin using EZ-link Sulfo-NHS Biotinylation kit (Perbio, UK) according to the manufacturer's instructions. A 96-well ELISA plate was coated with 0.4 µg ml^−1^ of unlabelled anti-SpyCEP antibody. Biotin labelled anti-SpyCEP antibody at 20 µg ml^−1^ was used to detect SpyCEP in cell-free culture supernatant and the concentration of SpyCEP was calculated against a standard curve based on recombinant SpyCEP (Turner *et al.*, 2009b). Samples were measured in duplicate by ELISA and data from the mean of three cultures were compared.

### Analysis of SpyCEP-cleaved CXC chemokines by SDS-PAGE and mass-spectrometry

Cell-free *S. pyogenes* culture supernatant was incubated at 37°C for ∼18 h with 200 ng carrier-free human/mouse chemokine (human CXCL1–CXCL12, mouse CXCL1/KC and CXCL2/MIP-2). Proteins were separated by pre-cast 12% Bis-Tris SDS-PAGE (Invitrogen) and stained with Colloidal Comassie Blue staining kit (Invitrogen). Exact cleavage sites were identified by mass spectrometry analysis as described previously for CXCL8/IL-8 and CXCL2/MIP-2 (Edwards *et al.*, 2005), except that cleaved proteins in solution were injected directly into an LTQ Linear IonTrap mass spectrometer (Thermo).

### Ethics statement

All animals were handled in strict accordance with good animal practice as defined by the UK Home Office, and all animal work was approved by the local ethics committee.

### Mouse infection models

CD1 female mice (6–8 weeks old) (Charles River, Margate, UK) were infected with 10^8^ cfu isogenic *S. pyogenes* or *L. lactis* directly into thigh muscle and quantitative end-points compared at 24 h. Mice were euthanized and blood was taken by cardiac puncture. To evaluate bacterial clearance, excised spleen, liver and infected thigh muscle from mice were individually weighed, homogenized, diluted and plated onto horse blood agar; and after overnight incubation at 37°C, cfu were noted. In some experiments using *L. lactis*, mice were euthanized at 3 and 6 h for histopathology. Survival end-points were defined by strict criteria relating to one or more of: loss of spontaneous movement, loss of response to stimuli, weight loss.

For intranasal infection mice were anesthetized with isoflurane and 10^8^ cfu *S. pyogenes* or 10^9^ cfu *L. lactis* were administered as 10 µl per nostril. After 24 h of infection, whole nasal tissue, NALT (homologue to human tonsil) and whole lung tissue were excised and bacterial load was quantified as above. Serum and infected tissue CXCL1/KC was measured by ELISA (R&D systems) that only detects full-length CXCL1/KC, not SpyCEP-cleaved CXCL1/KC. To maintain *L. lactis* constructs *in vivo*, both groups of *L. lactis*-infected mice were given three doses of 50 µg ml^−1^ erythromycin intraperitoneally; 4 h before infection then 6 h and 20 h post infection.

### Statistical analysis

Data were analysed using GraphPad Prism 4.0 (GraphPad software) and statistical significance determined between groups by applying the Mann–Whitney *U*-test. Values of *P* < 0.05 were considered as significant.
