# Supplementary material for: Chemokine-cleaving Streptococcus pyogenes protease SpyCEP is necessary and sufficient for bacterial dissemination within soft tissues and the respiratory tract
Source: Mol Microbiol. 2010 Feb 10;76(6):1387–97. doi: 10.1111/j.1365-2958.2010.07065.x (PMC2904501; doi:10.1111/j.1365-2958.2010.07065.x)
Supplement: Supplementary file 1 [file mmi0076-1387-SD1.pdf]

Supporting information

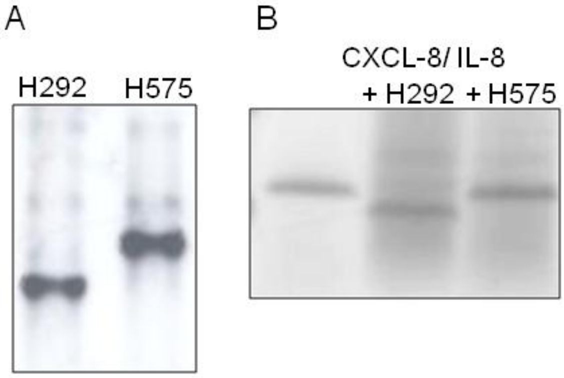

Figure S1. Confirmation of mutation in *S. pyogenes*. (A) Southern blot analysis of *Xmn*I-cut genomic DNA from isogenic *S. pyogenes* strains H292 (parent strain) and H575 (mutant strain) hybridized with DIG-labeled *cepA* PCR product. (B) Cleavage of IL-8 (CXCL8) by parent *S. pyogenes* strain H292 but not H575.
